# The Involvement of *ALPK3* in Hypertrophic Cardiomyopathy in East Asia

**DOI:** 10.3389/fmed.2022.915649

**Published:** 2022-06-15

**Authors:** Jiaqi Dai, Ke Li, Man Huang, Yang Sun, Hao Liu, Zongzhe Li, Peng Chen, Hong Wang, Dongyang Wu, Yanghui Chen, Lei Xiao, Haoran Wei, Rui Li, Liyuan Peng, Ting Yu, Yan Wang, Dao Wen Wang

**Affiliations:** ^1^Division of Cardiology, Department of Internal Medicine, Tongji Hospital, Tongji Medical College, Huazhong University of Science and Technology, Wuhan, China; ^2^Hubei Key Laboratory of Genetics and Molecular Mechanism of Cardiologic Disorders, Huazhong University of Science and Technology, Wuhan, China

**Keywords:** hypertrophic cardiomyopathy, East Asians, *ALPK3*, heterozygote, missense variant

## Abstract

**Objective:**

*ALPK3* is associated with a recessive form of pediatric cardiomyopathy accompanied by musculoskeletal and craniofacial abnormalities. Heterozygous truncating variants in this gene (*ALPK3*tv) have recently been confirmed as a cause of autosomal dominant hypertrophic cardiomyopathy (HCM). Whether *ALPK3* is also implicated in HCM in East Asia and the effect of missense variants in *ALPK3* on HCM remains unresolved.

**Methods:**

We compared the frequency of rare deleterious variants in *ALPK3* in a study cohort comprised of 793 HCM cases of East Asian descent to that in the controls subset of Genome Aggregation Database (gnomAD). Gene burden test was used to assess this association. The involvement of these variants in HCM was further validated by independent cohort. The clinical characteristics and prognoses of these carriers were compared with sarcomere-positive and negative patients.

**Results:**

Rare deleterious variants in *ALPK3* were significantly enriched in HCM compared with gnomAD controls (truncating: 4/793 vs. 4/4523, *P* = 0.02; missense: 25/793 vs. 46/4523, *P* = 2.56e-5). Replication in an independent cohort provided more supporting evidence. Further comparisons revealed that *ALPK3* carriers displayed more severe hypertrophy in interventricular septum (IVS) and apex, as well as greater maximal left ventricular wall thickness, relative to sarcomere negatives.

**Conclusion:**

Heterozygous rare variants in *ALPK3*, both missense and truncating variants, are associated with HCM in East Asians.

## Introduction

Hypertrophic cardiomyopathy (HCM) is the most common genetic heart disease characterized by left ventricular hypertrophy in the absence of abnormal loading conditions, affecting 0.16 to 0.29% of the general adult population (1). HCM is thought to be the most common cause of sudden cardiac death (SCD) in adolescents and young adults and is a major cause of heart failure, stroke, and ventricular arrhythmia (2). Given that approximately 30 to 60% of HCM is explained by pathogenic genetic variants in genes encoding proteins of the cardiac sarcomere, it was traditionally regarded as a monogenic disorder with an autosomal dominant pattern of inheritance (1). The missing causal gene of the remaining HCM cases remains largely unsolved.

*ALPK3* codes for alpha-protein kinase 3 and is involved in the differentiation and maturation of cardiomyocytes in mice (3). It was first reported in pediatric cardiomyopathy that patients with biallelic truncating genetic variants in *ALPK3* (*ALPK3*tv) displayed a phenotype of dilated cardiomyopathy (DCM) *in utero*, at birth, or early childhood, and often evolved to an HCM phenotype over time (4, 5). Subsequent studies emphasized the involvement of heterozygous truncating variants of *ALPK3* in HCM, where co-segregation of a heterozygous *ALPK3*tv with HCM was reported in a family and enrichment of *ALPK3*tv in HCM was observed in cardiomyopathy cohorts compared with the Genome Aggregation Database (gnomAD) population (5, 6). More recently, Lopes et al. (7) demonstrated that heterozygous *ALPK3*tv are disease-causing variants with autosomal dominant inheritance in ∼1.5% of HCM cases in an international cohort. In addition, a high proportion of implantable cardioverter–defibrillator (ICD) referrals and fibrosis was also observed in *ALPK3*tv carriers in this study. However, little is known about the role of rare deleterious missense variants in *ALPK3* in HCM. Whether *ALPK3tv* is also enriched in HCM patients in the East Asian population has not been closely examined. The prevalence of *ALPK3tv* carriers in East Asians and whether these cases are associated with the risk for SCD remains to be ascertained.

Thus, we compared the frequency of rare deleterious variants in *ALPK3* in our HCM cohort with whole-exome sequencing to that of the gnomAD control population using the gene burden test. These were missense and truncating variants. The association was established using an independent cohort. Moreover, these heterozygous *ALPK3* variant carriers were compared with non-carriers at baseline and follow-up to identify the clinical features of *ALPK3* carriers.

## Materials and Methods

### Study Cohort

The study cohort was comprised of 793 unrelated patients with HCM from 2007 to 2019, consecutively recruited from Tongji Hospital, Wuhan, China. The study was approved by the ethics committee of Tongji Hospital, and written informed consent was obtained from all the recruited patients. HCM was diagnosed as a maximal end-diastolic left ventricular (LV) wall thickness ≥ 15 mm on echocardiography or cardiac magnetic resonance images, in the absence of abnormal loading conditions or other cardiac or systemic diseases capable of producing the magnitude of hypertrophy. More limited hypertrophy (13–14 mm) was diagnosed in patients with a family history of HCM (1). Peripheral blood sample collection, DNA preparation, whole-exome sequencing, and variant calling were performed as previously described (8). For a case-control comparison, the *ALPK3* exome sequencing results of East Asian samples in the controls subset of gnomAD v2.1.1 (controls-only) were retrieved as normal controls (9). The total number of samples with high-quality genotypes was 4,523.

### Variant Annotation and Filtering

Variants in *ALPK3* were annotated according to the latest updated isoform (NM_020778.5; NP_065829.4) with ANNOVAR (10). Missense and truncating variants (frameshift, stop-gained, splice acceptor, and splice donor variants) in *ALPK3* with a minor allele frequency (MAF) < 0.0001 in all gnomAD sub-populations were regarded as rare and retained for subsequent burden tests. For missense variants, we further used REVEL and VEST3, which have been demonstrated to be the most accurate *in silico* functional prediction methods (11), to determine whether they are deleterious or not. All rare variants were confirmed using Sanger sequencing. Particularly, rare inframe insertion and deletion variants were excluded from the association analyses, for which pathogenicity was challenging to ascertain. As Supplementary Table 1 shows, inframe insertion/deletion variants in *ALPK3* were observed in two HCM cases and two controls, respectively.

### Follow-Up and Endpoint

Follow-ups with recruited patients were conducted annually through face-to-face visits and/or telephone contact until March 2019. The primary endpoint was death due to cardiovascular disease, including heart failure-related death and sudden death. Secondary outcomes included all-cause death, heart transplant, and progression to New York Heart Association (NYHA) class III/IV.

### Validation by Second Cohort

To replicate the findings observed in the study cohort, we enrolled another independent cohort from 2019 to 2020, which consisted of 419 patients diagnosed with HCM at Tongji Hospital, Wuhan, China. Whole-exome sequencing and variant filtering were performed according to the procedures described above.

### *ALPK3* Variants in Dilated Cardiomyopathy Cohort

To check whether *ALPK3* variants are also associated with DCM, we extracted *ALPK3* variants found in our previous DCM cohort, where whole-exome sequencing and variant calling was conducted in 1,041 DCM samples, as was done for HCM (12), and compared the prevalence of variants in the gnomAD controls.

### Transcriptome-Wide Association Study

Next, we integrated published GWAS results with transcriptomic prediction models to infer tissue-specific *ALPK3* expression, and then computed transcriptome-to-trait associations (13). Specifically, we first extracted GWAS summary statistics for HCM and a series of cardiac magnetic resonance imaging LV traits, including LV end-diastolic volume (LVEDV), body-surface-area (BSA) indexed LVEDV (LVEDVi), LV end-systolic volume (LVESV), BSA indexed LVESV (LVESVi), LV ejection fraction (LVEF), stroke volume (SV), BSA-indexed SV (SVi), LV mass (LVM), concentricity (LVM/LVEDV), mean LV wall thickness (meanWT), and global peak strain in radial, longitudinal, and circumferential directions from previously published studies (14–16). These summary statistics were then harmonized and imputed using 1000 Genomes reference data within a chromosomal region (Berisa–Pickrell linkage disequilibrium (LD) blocks). Harmonized and imputed GWAS summary statistics were used to perform TWAS for LV samples in GTEx v.8 eQTL data (17) with the MASHR-M model^1^.

### Gene-Based Burden Test and Statistical Analyses

The burden of rare variants in *ALPK3* was represented by the proportion of individuals carrying theses variants, and was compared between HCM cases and controls subset of gnomAD using Fisher’s exact test. Means for continuous variables were compared using the unpaired Student’s *t*-test, while frequencies of categorical variables were analyzed using the chi-square or Fisher’s exact test, as appropriate. All reported probabilities were two-sided and considered significant at *P* < 0.05.

## Results

The study cohort included 793 patients diagnosed with HCM, with a mean age of 52.64 ± 14.13 years at enrollment; 551 (69.5%) were male; all individuals were of East Asian descent (Supplementary Figure 1). Clinical information are shown in Table 1.

**TABLE 1:** Baseline comparisons of *ALPK3* carriers, sarcomere-positive, and sarcomere-negative patients.

	*ALPK3* ^+^	*ALPK3*^–^ Sarc^+^	*ALPK3*^–^ Sarc^–^	*P* value	
	(*N* = 28)	(*N* = 82)	(*N* = 621)	*ALPK3*^+^ vs. *ALPK3*^–^Sarc^+^	*ALPK3*^+^ vs. *ALPK3*^–^Sarc^–^
Gender = male (%)	16 (57.1)	54 (65.9)	439 (70.7)	0.408	0.126
Age of onset (years)	53.50 ± 17.21	45.17 ± 14.01	51.95 ± 14.20	0.012	0.576
Age at enrollment (years)	54.00 ± 17.18	48.00 ± 13.73	53.06 ± 14.17	0.064	0.733
Family history of HCM (%)	2 (7.1)	7 (8.5)	13 (2.1)	0.816	0.082
Family history of SCD (%)	0 (0.0)	2 (2.4)	3 (0.5)	0.404	0.712
Family history of stroke (%)	3 (10.7)	4 (4.9)	33 (5.3)	0.275	0.222
Smoke (%)	8 (28.6)	25 (30.5)	229 (36.9)	0.848	0.372
Alcohol intake (%)	3 (10.7)	19 (23.2)	158 (25.4)	0.155	0.078
NYHA heart function class (%)				0.078	0.093
I	3 (23.1)	5 (13.5)	69 (20.8)		
II	2 (15.4)	19 (51.4)	145 (43.8)		
III/IV	8 (61.5)	13 (35.1)	117 (35.3)		
Episode of syncope (%)	1 (3.6)	5 (6.1)	37 (6.0)	0.611	0.599
QTc (ms)	459.52 ± 36.63	460.90 ± 37.08	458.79 ± 42.13	0.869	0.938
QTc > 450 ms (%)	16 (59.3)	44 (61.1)	331 (55.3)	0.867	0.133
Atrial fibrillation (%)	7 (25.0)	19 (23.2)	77 (12.4)	0.844	0.052
Non-sustained ventricular tachycardia (%)	3 (10.7)	20 (24.4)	52 (8.4)	0.124	0.664
Atrioventricular block (%)	1 (3.6)	4 (4.9)	25 (4.0)	0.774	0.905
Any arrhythmia (%)	11 (39.3)	41 (50.0)	193 (31.1)	0.327	0.36
Non-fatal stroke (%)	6 (21.4)	3 (3.7)	80 (12.9)	0.003	0.192
CAD (%)	6 (21.4)	6 (7.3)	214 (34.5)	0.039	0.154
Diabetes mellitus (%)	3 (10.7)	7 (8.5)	128 (20.6)	0.729	0.202
Max wall thickness (mm)	19.36 ± 5.09	21.15 ± 5.18	17.16 ± 3.69	0.116	0.003
Max wall thickness ≥ 30 mm (%)	1 (3.6)	5 (6.1)	10 (1.6)	0.611	0.432
IVS (mm)	18.43 ± 5.57	20.50 ± 5.76	16.78 ± 3.88	0.101	0.032
LVPW (mm)	12.11 ± 3.29	11.06 ± 2.84	12.36 ± 3.05	0.109	0.664
Apex (mm)	11.89 ± 4.30	11.07 ± 3.32	10.84 ± 2.50	0.299	0.037
LAD (mm)	39.41 ± 5.63	42.00 ± 9.57	41.62 ± 7.59	0.186	0.135
LVEDD (mm)	46.22 ± 5.53	44.37 ± 7.82	48.67 ± 8.21	0.256	0.126
LVEF (%)	61.33 ± 9.21	63.84 ± 11.30	57.82 ± 13.23	0.299	0.172
LVOTG ≥ 30 mm Hg (%)	8 (28.6)	30 (36.6)	178 (28.7)	0.441	0.992
E/A	12.79 ± 36.66	6.49 ± 21.70	9.29 ± 28.96	0.291	0.545
E/E’	17.54 ± 7.06	16.61 ± 10.35	19.33 ± 10.02	0.693	0.397

*Values are N (%) or mean ± SD. ALPK3^+^, HCM cases carrying rare truncating or missense variants in ALPK3; Sarc^+^, sarcomere positive; Sarc**^–^**, sarcomere negative; SCD, sudden cardiac death; NYHA, New York Heart Association; CAD, coronary artery disease; IVS, interventricular septum; LVPW, left ventricular posterior wall; LAD, left atrial diameter; LVEDD, left ventricular end-diastolic dimension; LVEF, left ventricular ejection fraction; LVOTG, left ventricular outflow tract gradient.*

### Enrichment of *ALPK3* Rare Deleterious Variants in Hypertrophic Cardiomyopathy

**FIGURE 1:**
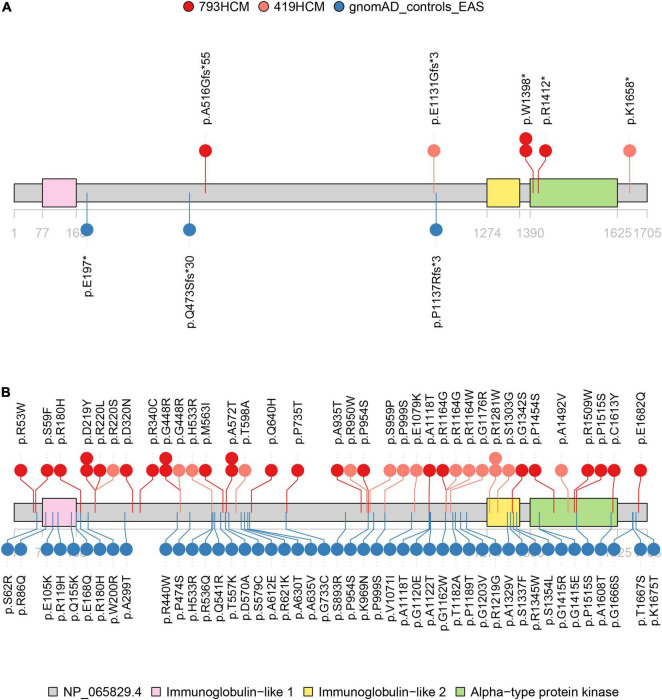
Distribution of rare variants in *ALPK3*. Structural and functional domains of *ALPK3* protein showing variant sites identified in study cohort (red), validation cohort (pink), and gnomAD controls (blue). **(A)** Distribution of rare truncating variants (frameshift and stop-gained). Another two splice site variants, respectively, observed in one HCM case (NM_020778.5: c.4410 + 1G > C) of the validation cohort and one control (NM_020778.5: c.4773-1G > C) were not displayed. **(B)** Distribution of rare missense variants.

**FIGURE 2:**
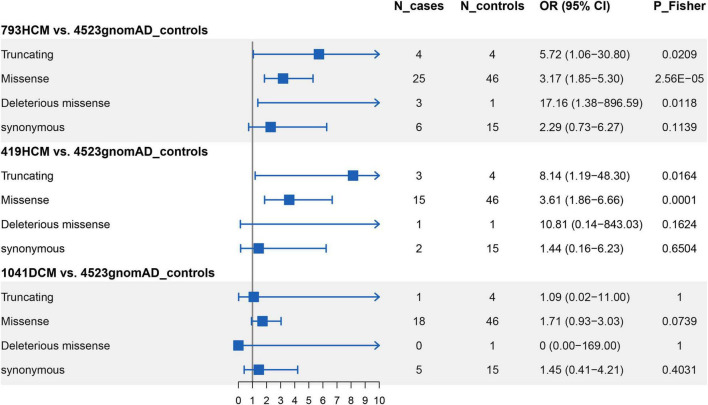
Gene-based burden tests for rare variants in *ALPK3*. N_cases: number of carries in cases; N_controls: number of carries in controls; OR: odds ratio; 95% CI: 95% confidence interval.

After screening for all exons of *ALPK3*, a total of 31 rare variants (MAF < 0.0001) across the *ALPK3* gene were identified in our study cohort, including six synonymous, 22 missense, one frameshift insertion, and two stop-gained variants (Supplementary Table 2). Among missense variants, three were predicted as deleterious by REVEL and VEST3. Following same filtering procedures, we also observed 65 rare variants of *ALPK3* in gnomAD controls (Supplementary Table 3). In total, 0.50% HCM cases (4/793) carried a rare truncating variant in *ALPK3* (Figure 1A), while 3.15% HCM cases (25/793) carried a rare missense variant (Figure 1B). In comparison, only 0.09% controls in gnomAD (4/4523) were heterozygous for rare truncating variants in *ALPK3*, and 1.02% controls (46/4523) were carriers of *ALPK3* rare missense variants. Further gene-based burden tests suggested that these *ALPK3* rare variants, both truncating and missense, were enriched in HCM compared with controls (truncating: 4/793 vs. 4/4523, *P* = 0.02; missense: 25/793 vs. 46/4523, *P* = 2.56e-5) (Figure 2). In particular, the prevalence of missense variants labeled as deleterious by REVEL and VEST3 remained significantly higher in HCM cases (3/793; 0.38%) than in controls (1/4523; 0.02%) with a *P* value of 0.01.

### Enrichment Validated by Second Cohort

To replicate the results found above, we performed whole-exome sequencing in another independent cohort comprised of 419 HCM patients. A total of 17 rare non-synonymous variants in *ALPK3* were identified in 18 HCM cases, including one frameshift deletion, one stop-gained, one splice site, and 14 missense variants (Supplementary Table 4). Once again, gene burden tests ascertained the association between *ALPK3* and HCM, where the prevalence of *ALPK3* rare variants in HCM was significantly higher than in gnomAD controls, when considering truncating variants (OR = 8.14, 95% CI: 1.19–48.30, *P* = 0.02) and missense variants (OR = 3.61, 95% CI: 1.86–6.66, *P* = 1.21e-4) separately. Moreover, meta-analysis combining the two cohorts reached same conclusions (truncating: 7/1212 vs. 4/4523, *P* = 0.002; missense: 40/1212 vs. 46/4523, *P* = 1.36e-7). Particularly, to examine the comparability, as well as potential systematic bias, between our HCM cohort and gnomAD controls, we further compared the burden of rare synonymous variants in *ALPK3* between the two groups and found no significant differences (793HCM vs. gnomAD controls: 6/793 vs. 15/4523, *P* = 0.11; 419HCM vs. gnomAD controls: 2/419 vs. 15/4523, *P* = 0.65) (Supplementary Table 5).

### Relation Between *ALPK3* and Dilated Cardiomyopathy

In our DCM cohort (*N* = 1,041), we found one rare splice site variant in a DCM case, and 17 missense variants in 18 DCM cases. As Supplementary Table 5 shows, neither truncating variants nor missense variants in *ALPK3* were associated with DCM.

### Clinical Characteristics of *ALPK3* Heterozygotes

To summarize the phenotypic features of *ALPK3* carriers, we compared the baseline statistics of patients carrying rare variants in *ALPK3* to those of sarcomere-positive and negative patients (Table 1). The most impressive characteristic was that *ALPK3* carriers presented with more severe hypertrophy in interventricular septum (IVS) (18.43 mm vs. 16.78 mm, *P* = 0.032) and apex (11.89 mm vs. 10.84 mm, *P* = 0.037), when compared to sarcomere negatives. Consistently, the maximal LV wall thickness was greater (19.36 mm vs. 17.16 mm, *P* = 0.003) in *ALPK3* carriers than in sarcomere negatives. In addition, when dividing *ALPK3* carriers into subgroups by variant type, no significant difference was found in the paired comparisons among carriers of *ALPK3* truncating variants, deleterious missense variants, and neutral missense variants, except that extreme LV hypertrophy (≥30 mm) appeared to be more prevalent in *ALPK3* truncating variant carriers than in neutral missense variant carriers (Supplementary Table 6).

### Outcomes of *ALPK3* Heterozygotes

In a total of 775 (97.7%) patients included in the final evaluation with a mean follow-up time of 32.78 ± 27.58 months, 5 (18.5%) carriers of rare *ALPK3* variants died, namely 1 SCD, 2 cardiac deaths, and 2 all-cause deaths.

### Association of *ALPK3* Expression With Left Ventricular Traits

**FIGURE 3:**
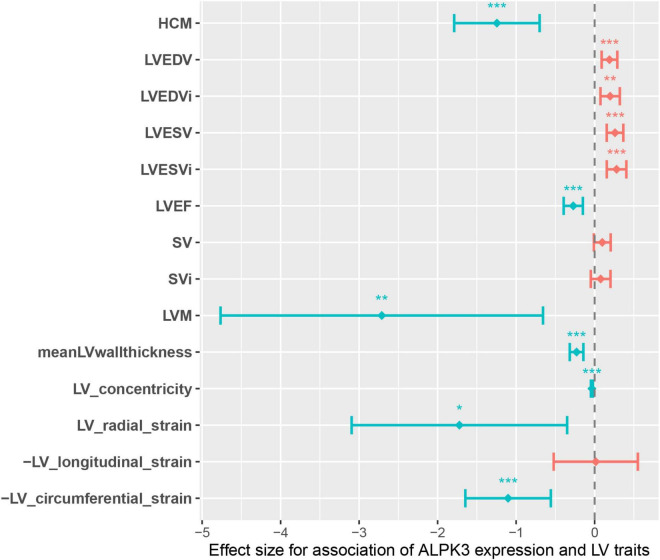
Genotype-inferred *ALPK3* expression-to-LV trait associations. Center values are the estimated association effect size and error bars indicate 95% confidence interval. Data shown correspond to that in Supplementary Table 7. LV, left ventricle/ventricular; LVEDV, LV end-diastolic volume; LVEDVi, body-surface-area (BSA) indexed LVEDV; LVESV, LV end-systolic volume; LVESVi, BSA indexed LVESV; LVEF, LV ejection fraction; SV, stroke volume; SVi, BSA indexed SV; LVM, LV mass; meanLVwallthickness, mean LV wall thickness. *P < 0.05, **P < 0.01, ***P < 0.001.

The causal effect of *ALPK3* truncating variants on the odds of HCM prompted us to hypothesized that *ALPK3* expression may be associated with the alteration of cardiac traits in general population. Therefore, we performed TWAS to estimate the involvement of *ALPK3* expression in HCM. We discovered that the predicted *ALPK3* expression in LV tissue was significantly different in HCM and several LV traits compared to control individuals. In summary, reduced expression of *ALPK3* was observed in HCM cases, as well as in individuals with decreased LVEDV, LVEDVi, LVESV, and LVESVi, and increased LVEF, LVM, meanWT, LV concentricity, and global LV strain measured in the radial and circumferential directions (Supplementary Table 7 and Figure 3). Taken together, these results suggested that *ALPK3* expression was associated with alteration of cardiac traits, and individuals with a decreased *ALPK3* expression have a higher risk for HCM and abnormal LV structure, which also adds to the causative role of truncating variants.

## Discussion

In this study, we performed exome sequencing of *ALPK3* in a study cohort comprised of 793 HCM cases with East Asian ancestry and identified a batch of rare variants in *ALPK3*. Gene-based burden tests suggested that both missense and truncating variants in *ALPK3* were enriched in HCM compared to gnomAD controls. Replication of these findings in another independent cohort with 419 HCM cases supported this association. Further baseline comparisons between carriers and sarcomere negatives indicated that *ALPK3* variants were associated with more severe hypertrophy in IVS and apex. TWAS using published GWAS and GTEx data, found evidence to support the causal effects of *ALPK3* expression on the odds of HCM.

Early studies focused more on biallelic *ALPK3*tv, which has been demonstrated to be associated with severe, childhood-onset cardiomyopathy and musculoskeletal abnormalities. Most *ALPK3*tv homozygotes die in the early years of life (4, 18–20). In our study and validation cohort, all *ALPK3* variant carriers were heterozygous, and none of them displayed an additional dysmorphic phenotype or had raised serum creatine kinase apart from adult-onset HCM. This is in accordance with a more recent publication, where no apparent musculoskeletal involvement, except for HCM, was found in a Thai family with a heterozygous *ALPK3*tv (6). Consistent with the data reported by Lopes et al. (7), heterozygous *ALPK3*tv was also enriched in our cohorts made up of East Asians. However, the prevalence of *ALPK3*tv observed in our cohort was much lower than that observed by Lopes et al. (0.58% vs. 1.56%, *P* = 0.03), especially compared to the South Asian population subgroup (0.58% vs. 5.68%, *P* = 6.89e-4). Another notable observation is that none of the *ALPK3*tv identified in our study was previously reported, together indicating a population-specific prevalence and distribution across the transcript of *ALPK3*tv.

Missense genetic variants in sarcomere genes account for the majority of HCM cases, which may alter protein structure and function by changing the amino acid composition of the encoded protein (2). For this reason, it is necessary to evaluate the effect of missense variants in *ALPK3* on HCM, whereas very little has been described in previous studies on this issue due to challenges in determining the pathogenicity of missense variants. Here, we attempted to identify rare deleterious missense variants using the *in silico* computational methods REVEL and VEST3. The two methods have been demonstrated to be effective with the best performance in predicting the pathogenicity of missense variants compared with other commonly used methods (11). Therefore, we additionally compared rare missense variants predicted as deleterious by both methods to minimize the risk of false positives between HCM cases and controls. Although these rare deleterious missense variants were no longer significantly enriched in the HCM validation cohort due to the limited number of carriers (Supplementary Table 5), the overall prevalence in the meta-analysis of the discovery and validation cohorts remained significantly higher relative to controls (4/1212 vs. 1/4523, *P* = 8.26e-3) with an OR of 14.96. The combination of these findings provides further support for the involvement of missense variants in *ALPK3* in HCM, although the causal effect remains to be confirmed.

It was observed that a high proportion of *ALPK3*tv carriers were judged to be at high risk of SCD by Lopes et al.; however, no arrhythmic events were observed to verify this correlation. In this study, we only observed 1 single incidence of SCD among the 27 *ALPK3* carriers that were followed, which was underpowered to draw any conclusion. Therefore, the link between *ALPK3* and SCD needs to be ascertained in larger cohorts. Given the greater maximum LV wall thickness in *ALPK3* variant carriers, it is reasonable to assume that *ALPK3* variants partially contribute to SCD through this risk factor. Therefore, it is clearly of urgent importance to focus research on the arrhythmogenic role of *ALPK3* variants. Preclinical evaluations in such patients are critical, particularly with respect to their capacity for early life-threatening arrhythmia. More aggressive strategies such as family screening and early arrhythmia monitoring should be recommended, as these patients may benefit from the primary prevention of SCD.

Recent GWAS have identified a locus near *ALPK3* that is significantly associated with HCM and DCM with opposite effects (16). Considering the haploinsufficiency caused by *ALPK3*tv, which serves as the main mechanism causing HCM, it could conceivably be hypothesized that a reduced dosage of the *ALPK3* transcript is causally associated with the risk of developing HCM. Our TWAS results supported this speculation. It is interesting to note that global alteration of LV traits associated with decreased *ALPK3* expression highly coincides with the representative morphological characteristics of HCM, including decreased LV volume, increased LV wall thickness and mass, and enhanced LV contractility. So we further speculated that any variant that could negatively alter the expression of the *ALPK3* protein may potentially contribute to HCM relevant phenotype, whether they are rare or common. On the contrary, it is unlikely that *ALPK3*tv could cause adult DCM, owing to the results that reduced *ALPK3* expression in left ventricle was associated with decreased LV volume (LVEDV, LVEDVi, LVESV, LVESVi) and increased LV contractility (LVEF, global strain in radial and circumferential directions). The observations in our DCM cohort also supported this point of view, where the frequency of *ALPK3*tv in DCM was similar to that in gnomAD controls.

### Study Limitations

The patients in our cohort were all recruited from a single center, which may limit the generalizability of the conclusions to other East Asian subgroups. The rare deleterious missense variants that carry a risk of false positives were selected using prediction tools and further studies on animal models and cardiomyocytes are needed to determine the pathogenicity of these variants.

## Conclusion

This study identified that heterozygous rare variants in *ALPK3*, both missense and truncating variants, were associated with an autosomal dominant HCM in East Asia *via* the demonstration of an enrichment of these variants in HCM cases. A considerable amount of work needs to be done to establish the causative role of *ALPK3* missense variants in HCM, as well as the arrhythmogenic effect of these variants.

## Data Availability Statement

The data presented in the study are deposited in the https://zenodo.org/record/6580063#.Yo4G__n1u2U, accession number doi: 10.5281/zenodo.6580063.

## Ethics Statement

The studies involving human participants were reviewed and approved by the Ethics Committee of Tongji Hospital, Wuhan, China. The patients/participants provided their written informed consent to participate in this study.

## Author Contributions

JD: conceptualization, formal analysis, investigation, methodology, visualization, and writing – original draft. KL and MH: data curation and investigation. YS: conceptualization, data curation, methodology, supervision, writing, review, and editing. HL: investigation. ZL and PC: conceptualization and methodology. HWa, DWu, YC, LX, and HWe: data curation. RL, LP, and TY: investigation. YW: project administration and supervision. DWa: conceptualization, funding acquisition, project administration, resources, supervision, writing, review, and editing. All authors contributed to the article and approved the submitted version.

## Conflict of Interest

The authors declare that the research was conducted in the absence of any commercial or financial relationships that could be construed as a potential conflict of interest.

## Publisher’s Note

All claims expressed in this article are solely those of the authors and do not necessarily represent those of their affiliated organizations, or those of the publisher, the editors and the reviewers. Any product that may be evaluated in this article, or claim that may be made by its manufacturer, is not guaranteed or endorsed by the publisher.

## Footnotes

1
https://github.com/hakyimlab/MetaXcan/wiki
