# Supplementary material for: The Involvement of ALPK3 in Hypertrophic Cardiomyopathy in East Asia
Source: Front Med (Lausanne). 2022 Jun 15;9:915649. doi: 10.3389/fmed.2022.915649 (PMC9240616; doi:10.3389/fmed.2022.915649)
Supplement: Supplementary file 1 [file Data_Sheet_1.DOCX]

SUPPLEMENTARY MATERIAL

**The involvement of *ALPK3* in hypertrophic cardiomyopathy in East Asia**

Jiaqi Dai^1,2^, MD; Ke Li^2^, MD; Man Huang^2^, MD; Yang Sun^1,2^, MD, PhD; Hao Liu^2^, MD; Zongzhe Li^1,2^, MD, PhD; Peng Chen^1,2^, MD, PhD; Hong Wang^1^, PhD; Dongyang Wu^2^, MD; Yanghui Chen^2^, MD; Lei Xiao^2^, MD; Haoran Wei^2^, MD; Rui Li^1,2^, MD, PhD; Liyuan Peng ^1^, MD, PhD; Ting Yu^1^, BN; Yan Wang^1,2^, MD, PhD and Dao Wen Wang^1,2^, MD, PhD

**Supplementary Methods**

**Supplementary Figure 1. Check for population stratification among samples.**

**Supplementary Table 1. Rare inframe insertion and deletion variants of *ALPK3* observed in our HCM cases and controls.**

**Supplementary Table 2. Rare variants of *ALPK3* identified in 793 cases with HCM.**

**Supplementary Table 3. Supplementary Table 2. Rare variants of *ALPK3* in East Asian samples of gnomAD v2.1.1 (controls subset).**

**Supplementary Table 4. Rare variants of *ALPK3* identified in 419 cases with HCM.**

**Supplementary Table 5 Gene-based burden tests for rare variants in *ALPK3*.**

**Supplementary Table 6. Baseline comparisons among individuals carrying rare truncating and missense variants in *ALPK3*.**

**Supplementary Table 7. Genotype-inferred *ALPK3* expression-to-LV trait associations.**

**Supplementary Methods**

*Comparisons among ALPK3 carriers, sarcomere-positive, and sarcomere-negative patients*

The pathogenicity of variant was determined by American College of Medical Genetics and Genomics (ACMG). Individuals were assigned sarcomere-positive status if they harbored a variant classified as likely pathogenic or pathogenic across 8 well-established sarcomere genes (*MYBPC3*, *ACTC1*, *MYH7*, *MYL2*, *MYL3*, *TNNT2*, *TNNI3* and *TPM1*), and those with no pathogenic/likely pathogenic variants were labeled as sarcomere negatives. Individuals who harbored variants of uncertain significance (VUSs) and no pathogenic/likely pathogenic variant were excluded.


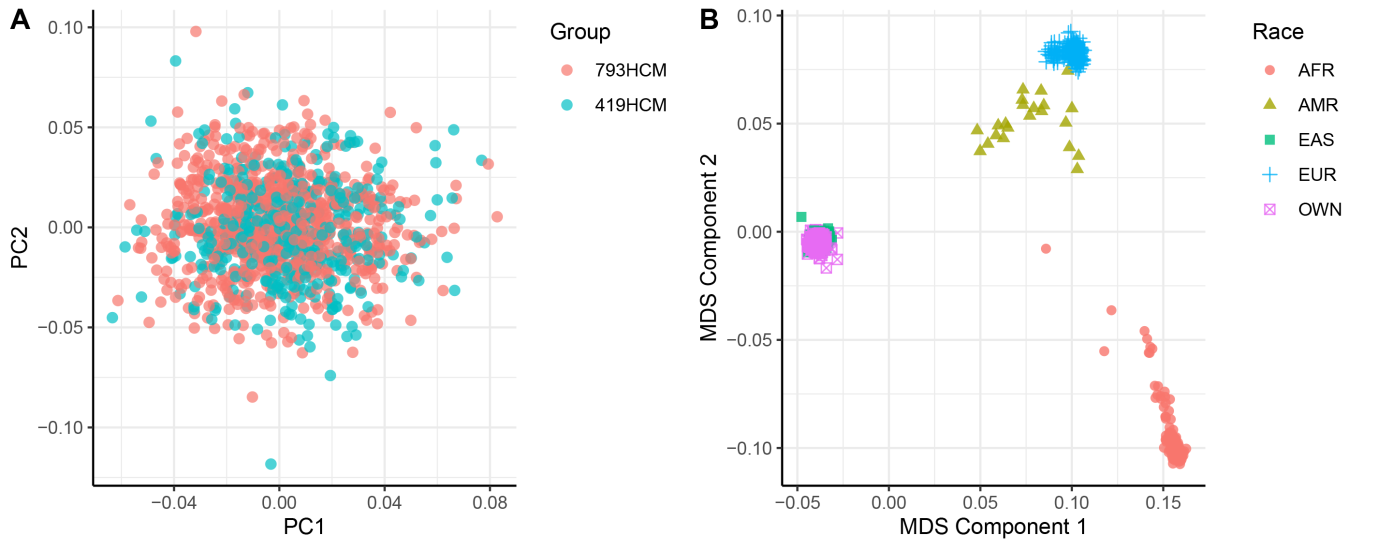


**Supplementary Figure 1. Check for population stratification among samples. (A)** Results of principle component analysis (PCA). The graph includes 793 HCM cases from study cohort and 419 HCM cases from validation cohort. The PCA revealed the homogenous population structure between the two cohorts. **(B)** Multidimensional scaling (MDS) analysis of genetic race using 1000 Genomes data as anchors.

| **Supplementary Table 1. Rare inframe insertion and deletion variants of *ALPK3* observed in our HCM cases and controls.** | | | | | | | | | | |
| --- | --- | --- | --- | --- | --- | --- | --- | --- | --- | --- |
| Chromsome | Position | Consequence | HGVS.c | HGVS.p | AF_gnomAD | AF_gnomAD_EAS | AF_gnomAD_popmax | Predicted effect by REVEL and VEST3 | N_carriers |  |
| 15 | 85382255 | inframe_deletion | c.350_352del | p.Leu117_Asp118delinsHis | 0 | 0 | 0 | - | 1 case (793HCM) |  |
| 15 | 85399823 | inframe_deletion | c.1862_1879del | p.Arg621_Gly626del | 0 | 0 | 0 | - | 1 case (793HCM) |  |
| 15 | 85370783 | inframe_deletion | c.256_267del | p.Arg86_Ser89del | 3.98E-06 | 5.44E-05 | 5.44E-05 | - | 1 control  (gnomAD) |  |
| 15 | 85411569 | inframe_insertion | c.5002_5003insTAGTCC | p.Thr1667_Arg1668insLeuVal | 3.98E-06 | 5.44E-05 | 5.44E-05 | - | 1 control  (gnomAD) |  |
| Abbreviations: HGVS.c: Variant using HGVS notation (DNA level); HGVS.p: HGVS notation (protein level); AF_gnomAD, Alternate allele frequency in samples of gnomAD v2.1.1 data set; AF_gnomAD_EAS, Alternate allele frequency in samples of East Asian ancestry of gnomAD; AF_gnomAD_popmax, Maximum allele frequency across populations in gnomAD; N_carriers, Number of individuals carrying the variant. | | | | | | | | | |  |
|  |  |  |  |  |  |  |  |  |  |  |
|  |  |  |  |  |  |  |  |  |  |  |
|  |  |  |  |  |  |  |  |  |  |  |
|  |  |  |  |  |  |  |  |  |  |  |

| **Supplementary Table 2. Rare variants of *ALPK3* identified in 793 cases with HCM.** | | | | | | | | | |
| --- | --- | --- | --- | --- | --- | --- | --- | --- | --- |
| Chromsome | Position | Consequence | HGVS.c | HGVS.p | AF_gnomAD | AF_gnomAD_EAS | AF_gnomAD_popmax | Predicted effect by REVEL and VEST3 | N_carriers |
| 15 | 85366574 | missense | c.157C>T | p.Arg53Trp | 7.95E-06 | 5.44E-05 | 5.44E-05 | neutral | 1 |
| 15 | 85366593 | missense | c.176C>T | p.Ser59Phe | 0 | 0 | 0 | neutral | 1 |
| 15 | 85383049 | missense | c.539G>A | p.Arg180His | 3.98E-06 | 5.44E-05 | 5.44E-05 | neutral | 1 |
| 15 | 85383165 | missense | c.655G>T | p.Asp219Tyr | 4.01E-06 | 5.45E-05 | 5.45E-05 | neutral | 2 |
| 15 | 85383169 | missense | c.659G>T | p.Arg220Leu | 0 | 0 | 0 | deleterious | 1 |
| 15 | 85383468 | missense | c.958G>A | p.Asp320Asn | 0 | 0 | 0 | neutral | 1 |
| 15 | 85383528 | missense | c.1018C>T | p.Arg340Cys | 0 | 0 | 0 | neutral | 1 |
| 15 | 85383641 | synonymous | c.1131G>T | p.Gly377%3D | 0 | 0 | 0 | - | 1 |
| 15 | 85383833 | synonymous | c.1323G>A | p.Ala441%3D | 9.94E-06 | 0 | 2.12E-05 | - | 1 |
| 15 | 85383852 | missense | c.1342G>A | p.Gly448Arg | 0 | 0 | 0 | neutral | 2 |
| 15 | 85384051 | frameshift | c.1544dup | p.Ala516GlyfsTer55 | 0 | 0 | 0 | - | 1 |
| 15 | 85399658 | missense | c.1689G>A | p.Met563Ile | 0 | 0 | 0 | neutral | 1 |
| 15 | 85399683 | missense | c.1714G>A | p.Ala572Thr | 3.98E-06 | 5.44E-05 | 5.44E-05 | neutral | 2 |
| 15 | 85399889 | missense | c.1920G>C | p.Gln640His | 0 | 0 | 0 | neutral | 1 |
| 15 | 85400172 | missense | c.2203C>A | p.Pro735Thr | 0 | 0 | 0 | neutral | 1 |
| 15 | 85400642 | synonymous | c.2673C>T | p.His891%3D | 1.59E-05 | 5.44E-05 | 6.17E-05 | - | 1 |
| 15 | 85400744 | synonymous | c.2775A>G | p.Pro925%3D | 0 | 0 | 0 | - | 1 |
| 15 | 85400772 | missense | c.2803G>A | p.Ala935Thr | 0 | 0 | 0 | neutral | 1 |
| 15 | 85400829 | missense | c.2860C>T | p.Pro954Ser | 8.95E-06 | 5.53E-05 | 5.53E-05 | neutral | 1 |
| 15 | 85401050 | synonymous | c.3081A>G | p.Ala1027%3D | 4.02E-06 | 0 | 8.93E-06 | - | 1 |
| 15 | 85401321 | missense | c.3352G>A | p.Ala1118Thr | 4.99E-06 | 5.98E-05 | 5.98E-05 | neutral | 1 |
| 15 | 85401459 | missense | c.3490C>G | p.Arg1164Gly | 4.15E-06 | 0 | 2.95E-05 | neutral | 1 |
| 15 | 85403065 | missense | c.4024G>A | p.Gly1342Ser | 1.99E-05 | 0 | 9.80E-05 | deleterious | 1 |
| 15 | 85405930 | stop_gained | c.4194G>A | p.Trp1398Ter | 0 | 0 | 0 | - | 2 |
| 15 | 85405970 | stop_gained | c.4234C>T | p.Arg1412Ter | 3.98E-06 | 5.44E-05 | 5.44E-05 | - | 1 |
| 15 | 85406096 | missense | c.4360C>T | p.Pro1454Ser | 7.96E-06 | 5.44E-05 | 5.44E-05 | neutral | 1 |
| 15 | 85407698 | missense | c.4525C>T | p.Arg1509Trp | 4.38E-05 | 0 | 8.79E-05 | neutral | 1 |
| 15 | 85407716 | missense | c.4543C>T | p.Pro1515Ser | 3.98E-06 | 5.44E-05 | 5.44E-05 | neutral | 1 |
| 15 | 85411407 | missense | c.4838G>A | p.Cys1613Tyr | 0 | 0 | 0 | deleterious | 1 |
| 15 | 85411600 | synonymous | c.5031C>T | p.Thr1677%3D | 3.98E-06 | 5.44E-05 | 5.44E-05 | - | 1 |
| 15 | 85411613 | missense | c.5044G>C | p.Glu1682Gln | 0 | 0 | 0 | neutral | 1 |
| Abbreviations: HGVS.c: Variant using HGVS notation (DNA level); HGVS.p: HGVS notation (protein level); AF_gnomAD, Alternate allele frequency in samples of gnomAD v2.1.1 data set; AF_gnomAD_EAS, Alternate allele frequency in samples of East Asian ancestry of gnomAD; AF_gnomAD_popmax, Maximum allele frequency across populations in gnomAD; N_carriers, Number of individuals carrying the variant. | | | | | | | | | |
|  |  |  |  |  |  |  |  |  |  |
|  |  |  |  |  |  |  |  |  |  |
|  |  |  |  |  |  |  |  |  |  |

| **Supplementary Table 3. Rare variants of *ALPK3* in East Asian samples of gnomAD v2.1.1 (controls subset).** | | | | | | | | | |
| --- | --- | --- | --- | --- | --- | --- | --- | --- | --- |
| Chromsome | Position | Consequence | HGVS.c | HGVS.p | AF_gnomAD | AF_gnomAD_EAS | AF_gnomAD_popmax | Predicted effect by REVEL and VEST3 | N_carriers |
| 15 | 85370718 | missense | c.186C>A | p.Ser62Arg | 7.96E-06 | 5.44E-05 | 5.44E-05 | neutral | 1 |
| 15 | 85370763 | synonymous | c.231G>A | p.Pro77%3D | 1.19E-05 | 5.44E-05 | 6.15E-05 | - | 1 |
| 15 | 85370789 | missense | c.257G>A | p.Arg86Gln | 2.39E-05 | 5.44E-05 | 8.67E-05 | neutral | 1 |
| 15 | 85370799 | synonymous | c.267C>T | p.Ser89%3D | 2.79E-05 | 5.44E-05 | 6.53E-05 | - | 1 |
| 15 | 85382219 | missense | c.313G>A | p.Glu105Lys | 4.00E-06 | 5.44E-05 | 5.44E-05 | neutral | 1 |
| 15 | 85382262 | missense | c.356G>A | p.Arg119His | 8.02E-06 | 5.45E-05 | 5.45E-05 | neutral | 1 |
| 15 | 85382278 | synonymous | c.372A>C | p.Pro124%3D | 4.06E-06 | 5.47E-05 | 5.47E-05 | - | 1 |
| 15 | 85382933 | synonymous | c.423G>A | p.Arg141%3D | 4.15E-06 | 5.46E-05 | 5.46E-05 | - | 1 |
| 15 | 85382972 | synonymous | c.462C>T | p.Ala154%3D | 3.99E-06 | 5.44E-05 | 5.44E-05 | - | 1 |
| 15 | 85382973 | missense | c.463C>A | p.Gln155Lys | 3.99E-06 | 5.44E-05 | 5.44E-05 | neutral | 1 |
| 15 | 85383012 | missense | c.502G>C | p.Glu168Gln | 3.98E-06 | 5.44E-05 | 5.44E-05 | neutral | 1 |
| 15 | 85383049 | missense | c.539G>A | p.Arg180His | 3.98E-06 | 5.44E-05 | 5.44E-05 | neutral | 1 |
| 15 | 85383099 | stop_gained | c.589G>T | p.Glu197Ter | 3.98E-06 | 5.44E-05 | 5.44E-05 | - | 1 |
| 15 | 85383108 | missense | c.598T>C | p.Trp200Arg | 3.98E-06 | 5.44E-05 | 5.44E-05 | neutral | 1 |
| 15 | 85383161 | synonymous | c.651C>T | p.Ser217%3D | 4.00E-06 | 5.45E-05 | 5.45E-05 | - | 1 |
| 15 | 85383356 | synonymous | c.846C>T | p.Pro282%3D | 1.59E-05 | 5.44E-05 | 6.53E-05 | - | 1 |
| 15 | 85383405 | missense | c.895G>A | p.Ala299Thr | 3.99E-06 | 5.44E-05 | 5.44E-05 | neutral | 1 |
| 15 | 85383828 | missense | c.1318C>T | p.Arg440Trp | 9.84E-06 | 5.91E-05 | 5.91E-05 | neutral | 1 |
| 15 | 85383921 | frameshift | c.1417del | p.Gln473SerfsTer30 | 2.05E-05 | 5.47E-05 | 5.47E-05 | - | 1 |
| 15 | 85383930 | missense | c.1420C>T | p.Pro474Ser | 4.04E-06 | 5.46E-05 | 5.46E-05 | neutral | 1 |
| 15 | 85384108 | missense | c.1598A>G | p.His533Arg | 2.41E-05 | 5.45E-05 | 5.45E-05 | neutral | 1 |
| 15 | 85384117 | missense | c.1607G>A | p.Arg536Gln | 4.04E-06 | 5.48E-05 | 5.48E-05 | neutral | 1 |
| 15 | 85384132 | missense | c.1622A>G | p.Gln541Arg | 4.12E-06 | 5.54E-05 | 5.54E-05 | neutral | 1 |
| 15 | 85399639 | missense | c.1670C>A | p.Thr557Lys | 4.23E-06 | 5.55E-05 | 5.55E-05 | neutral | 1 |
| 15 | 85399646 | synonymous | c.1677G>A | p.Thr559%3D | 8.23E-06 | 5.50E-05 | 0.000055 | - | 1 |
| 15 | 85399678 | missense | c.1709A>C | p.Asp570Ala | 3.99E-06 | 5.44E-05 | 5.44E-05 | neutral | 1 |
| 15 | 85399705 | missense | c.1736C>G | p.Ser579Cys | 3.98E-06 | 5.44E-05 | 5.44E-05 | neutral | 1 |
| 15 | 85399804 | missense | c.1835C>A | p.Ala612Glu | 3.98E-06 | 5.44E-05 | 5.44E-05 | neutral | 1 |
| 15 | 85399831 | missense | c.1862G>A | p.Arg621Lys | 3.98E-06 | 5.44E-05 | 5.44E-05 | neutral | 1 |
| 15 | 85399857 | missense | c.1888G>A | p.Ala630Thr | 3.98E-06 | 5.44E-05 | 5.44E-05 | neutral | 1 |
| 15 | 85399873 | missense | c.1904C>T | p.Ala635Val | 3.98E-06 | 5.44E-05 | 5.44E-05 | neutral | 1 |
| 15 | 85400114 | synonymous | c.2145C>T | p.Ser715%3D | 7.96E-06 | 5.44E-05 | 5.44E-05 | - | 1 |
| 15 | 85400166 | missense | c.2197G>T | p.Gly733Cys | 3.98E-06 | 5.44E-05 | 5.44E-05 | neutral | 1 |
| 15 | 85400567 | synonymous | c.2598G>T | p.Thr866%3D | 3.98E-06 | 5.44E-05 | 5.44E-05 | - | 1 |
| 15 | 85400646 | missense | c.2677A>C | p.Ser893Arg | 3.99E-06 | 5.44E-05 | 5.44E-05 | neutral | 1 |
| 15 | 85400829 | missense | c.2860C>T | p.Pro954Ser | 8.95E-06 | 5.53E-05 | 5.53E-05 | neutral | 1 |
| 15 | 85400876 | missense | c.2907G>T | p.Lys969Asn | 4.14E-06 | 5.50E-05 | 0.000055 | neutral | 1 |
| 15 | 85400964 | missense | c.2995C>T | p.Pro999Ser | 4.00E-06 | 5.45E-05 | 5.45E-05 | neutral | 1 |
| 15 | 85401086 | synonymous | c.3117C>T | p.Arg1039%3D | 4.06E-06 | 5.49E-05 | 5.49E-05 | - | 1 |
| 15 | 85401180 | missense | c.3211G>A | p.Val1071Ile | 4.23E-06 | 5.65E-05 | 5.65E-05 | neutral | 1 |
| 15 | 85401321 | missense | c.3352G>A | p.Ala1118Thr | 4.99E-06 | 5.98E-05 | 5.98E-05 | neutral | 1 |
| 15 | 85401328 | missense | c.3359G>A | p.Gly1120Glu | 4.97E-06 | 5.99E-05 | 5.99E-05 | neutral | 1 |
| 15 | 85401333 | missense | c.3364G>A | p.Ala1122Thr | 4.94E-06 | 5.98E-05 | 5.98E-05 | neutral | 1 |
| 15 | 85401353 | synonymous | c.3384C>T | p.Pro1128%3D | 4.60E-06 | 5.77E-05 | 5.77E-05 | - | 1 |
| 15 | 85401376 | frameshift | c.3409_3436del | p.Pro1137ArgfsTer3 | 4.23E-06 | 5.58E-05 | 5.58E-05 | - | 1 |
| 15 | 85401453 | missense | c.3484G>T | p.Gly1162Trp | 4.11E-06 | 5.58E-05 | 5.58E-05 | deleterious | 1 |
| 15 | 85401513 | missense | c.3544A>G | p.Thr1182Ala | 5.07E-06 | 6.97E-05 | 6.97E-05 | neutral | 1 |
| 15 | 85401534 | missense | c.3565C>A | p.Pro1189Thr | 1.14E-05 | 7.88E-05 | 7.88E-05 | neutral | 1 |
| 15 | 85401577 | missense | c.3608G>T | p.Gly1203Val | 6.27E-06 | 8.56E-05 | 8.56E-05 | neutral | 1 |
| 15 | 85401624 | missense | c.3655A>G | p.Arg1219Gly | 1.25E-05 | 8.19E-05 | 8.19E-05 | neutral | 1 |
| 15 | 85403027 | missense | c.3986C>T | p.Ala1329Val | 5.23E-05 | 5.45E-05 | 0.000098 | neutral | 1 |
| 15 | 85403051 | missense | c.4010C>T | p.Ser1337Phe | 3.99E-06 | 5.44E-05 | 5.44E-05 | neutral | 1 |
| 15 | 85403074 | missense | c.4033C>T | p.Arg1345Trp | 7.17E-05 | 5.44E-05 | 5.44E-05 | neutral | 1 |
| 15 | 85403102 | missense | c.4061C>T | p.Ser1354Leu | 5.17E-05 | 5.44E-05 | 6.53E-05 | neutral | 1 |
| 15 | 85405979 | missense | c.4243G>A | p.Gly1415Arg | 3.98E-06 | 5.44E-05 | 5.44E-05 | neutral | 1 |
| 15 | 85405980 | missense | c.4244G>A | p.Gly1415Glu | 3.98E-06 | 5.44E-05 | 5.44E-05 | neutral | 1 |
| 15 | 85405990 | synonymous | c.4254T>C | p.Cys1418%3D | 3.98E-06 | 5.44E-05 | 5.44E-05 | - | 1 |
| 15 | 85407716 | missense | c.4543C>T | p.Pro1515Ser | 3.98E-06 | 5.44E-05 | 5.44E-05 | neutral | 1 |
| 15 | 85411341 | splice_acceptor | c.4773-1G>C | - | 4.05E-06 | 5.45E-05 | 5.45E-05 | - | 1 |
| 15 | 85411391 | missense | c.4822G>A | p.Ala1608Thr | 3.99E-06 | 5.44E-05 | 5.44E-05 | neutral | 1 |
| 15 | 85411429 | synonymous | c.4860G>A | p.Leu1620%3D | 3.98E-06 | 5.44E-05 | 5.44E-05 | - | 1 |
| 15 | 85411565 | missense | c.4996G>A | p.Gly1666Ser | 3.98E-06 | 5.44E-05 | 5.44E-05 | neutral | 1 |
| 15 | 85411568 | missense | c.4999A>T | p.Thr1667Ser | 3.98E-06 | 5.44E-05 | 5.44E-05 | neutral | 1 |
| 15 | 85411593 | missense | c.5024A>C | p.Lys1675Thr | 3.98E-06 | 5.44E-05 | 5.44E-05 | neutral | 1 |
| 15 | 85411600 | synonymous | c.5031C>T | p.Thr1677%3D | 3.98E-06 | 5.44E-05 | 5.44E-05 | - | 1 |
| Abbreviations: HGVS.c: Variant using HGVS notation (DNA level); HGVS.p: HGVS notation (protein level); AF_gnomAD, Alternate allele frequency in samples of gnomAD v2.1.1 data set; AF_gnomAD_EAS, Alternate allele frequency in samples of East Asian ancestry of gnomAD; AF_gnomAD_popmax, Maximum allele frequency across populations in gnomAD; N_carriers, Number of individuals carrying the variant. | | | | | | | | | |
|  |  |  |  |  |  |  |  |  |  |
|  |  |  |  |  |  |  |  |  |  |
|  |  |  |  |  |  |  |  |  |  |
|  |  |  |  |  |  |  |  |  |  |

| **Supplementary Table 4. Rare variants of *ALPK3* identified in 419 cases with HCM.** | | | | | | | | | |
| --- | --- | --- | --- | --- | --- | --- | --- | --- | --- |
| Chromsome | Position | Consequence | HGVS.c | HGVS.p | AF_gnomAD | AF_gnomAD_EAS | AF_gnomAD_popmax | Predicted effect by REVEL and VEST3 | N_carriers |
| 15 | 85383168 | missense | c.658C>A | p.Arg220Ser | 0 | 0 | 0 | neutral | 1 |
| 15 | 85383852 | missense | c.1342G>A | p.Gly448Arg | 0 | 0 | 0 | neutral | 1 |
| 15 | 85384108 | missense | c.1598A>G | p.His533Arg | 2.41E-05 | 5.45E-05 | 5.45E-05 | neutral | 1 |
| 15 | 85399761 | missense | c.1792A>G | p.Thr598Ala | 0 | 0 | 0 | neutral | 1 |
| 15 | 85400105 | synonymous | c.2136A>C | p.Ser712%3D | 0 | 0 | 0 | - | 1 |
| 15 | 85400817 | missense | c.2848C>T | p.Arg950Trp | 2.28E-05 | 0 | 8.67E-05 | neutral | 1 |
| 15 | 85400844 | missense | c.2875T>C | p.Ser959Pro | 0 | 0 | 0 | neutral | 1 |
| 15 | 85400964 | missense | c.2995C>T | p.Pro999Ser | 4.00E-06 | 5.45E-05 | 5.45E-05 | neutral | 1 |
| 15 | 85401204 | missense | c.3235G>A | p.Glu1079Lys | 0 | 0 | 0 | neutral | 1 |
| 15 | 85401360 | frameshift | c.3392del | p.Glu1131GlyfsTer3 | 0 | 0 | 0 | - | 1 |
| 15 | 85401459 | missense | c.3490C>G | p.Arg1164Gly | 4.15E-06 | 0 | 2.95E-05 | neutral | 1 |
| 15 | 85401459 | missense | c.3490C>T | p.Arg1164Trp | 8.29E-06 | 5.63E-05 | 5.63E-05 | deleterious | 1 |
| 15 | 85401495 | missense | c.3526G>A | p.Gly1176Arg | 0 | 0 | 0 | neutral | 1 |
| 15 | 85402497 | missense | c.3841C>T | p.Arg1281Trp | 2.78E-05 | 0 | 6.16E-05 | neutral | 2 |
| 15 | 85402563 | missense | c.3907A>G | p.Ser1303Gly | 0 | 0.00E+00 | 0 | neutral | 1 |
| 15 | 85406026 | synonymous | c.4290C>T | p.Tyr1430%3D | 3.58E-05 | 5.44E-05 | 6.16E-05 | - | 1 |
| 15 | 85406147 | splice_donor | c.4410+1G>C | - | 0 | 0 | 0 | - | 1 |
| 15 | 85406847 | missense | c.4475C>T | p.Ala1492Val | 0 | 0 | 0 | neutral | 1 |
| 15 | 85411541 | stop_gained | c.4972A>T | p.Lys1658Ter | 0 | 0 | 0 | - | 1 |
| Abbreviations: HGVS.c: Variant using HGVS notation (DNA level); HGVS.p: HGVS notation (protein level); AF_gnomAD, Alternate allele frequency in samples of gnomAD v2.1.1 data set; AF_gnomAD_EAS, Alternate allele frequency in samples of East Asian ancestry of gnomAD; AF_gnomAD_popmax, Maximum allele frequency across populations in gnomAD; N_carriers, Number of individuals carrying the variant. | | | | | | | | | |
|  |  |  |  |  |  |  |  |  |  |
|  |  |  |  |  |  |  |  |  |  |
|  |  |  |  |  |  |  |  |  |  |
|  |  |  |  |  |  |  |  |  |  |

| **Supplementary Table 5. Gene-based burden tests for rare variants in *ALPK3*.** | | | | |
| --- | --- | --- | --- | --- |
|  | N_cases | N_controls | OR (95% CI) | P_Fisher |
| 793HCM vs. 4523gnomAD_controls |  |  |  |  |
| Truncating | 4 | 4 | 5.72 (1.06-30.80) | 0.02086 |
| Missense | 25 | 46 | 3.17 (1.85-5.30) | 0.00002561 |
| Deleterious missense | 3 | 1 | 17.16 (1.38-896.59) | 0.01176 |
| synonymous | 6 | 15 | 2.29 (0.73-6.27) | 0.1139 |
| 419HCM vs. 4523gnomAD_controls |  |  |  |  |
| Truncating | 3 | 4 | 8.14 (1.19-48.30) | 0.01635 |
| Missense | 15 | 46 | 3.61 (1.86-6.66) | 0.0001206 |
| Deleterious missense | 1 | 1 | 10.81 (0.14-843.03) | 0.1624 |
| synonymous | 2 | 15 | 1.44 (0.16-6.23) | 0.6504 |
| 1041DCM vs. 4523gnomAD_controls |  |  |  |  |
| Truncating | 1 | 4 | 1.09 (0.02-11.00) | 1 |
| Missense | 18 | 46 | 1.71 (0.93-3.03) | 0.07386 |
| Deleterious missense | 0 | 1 | 0 (0.00-169.00) | 1 |
| synonymous | 5 | 15 | 1.45 (0.41-4.21) | 0.4031 |
| Abbreviations: N_cases, number of carriers in cases; N_controls, number of carriers in controls; OR, odds ratio; 95%CI, 95% confidence interval. | | | | |
|  |  |  |  |  |
|  |  |  |  |  |

| **Supplementary Table 6. Baseline comparisons among individuals carrying rare truncating and missense variants in *ALPK3*.** | | | | | | |  |  |
| --- | --- | --- | --- | --- | --- | --- | --- | --- |
|  | ***ALPK3tv*** | ***ALPK3dmv*** | ***ALPK3nmv*** | ***P* value** | | | | |
|  | (*N* = 4) | (*N* = 3) | (*N* = 21) | *ALPK3tv* *vs.* *ALPK3dmv* | *ALPK3tv* *vs.* *ALPK3nmv* | *ALPK3dmv* *vs.* *ALPK3nmv* | |  |
| Gender =male (%) | 1 (25.0) | 2 (66.7) | 13 (61.9) | 0.27 | 0.173 | 0.873 | |  |
| Age of onset (years) | 46.00 ± 23.62 | 51.00 ± 6.24 | 55.29 ± 17.22 | 0.741 | 0.359 | 0.678 | |  |
| Age at enrolment (years) | 46.25 ± 23.92 | 51.33 ± 6.66 | 55.86 ± 17.07 | 0.74 | 0.341 | 0.659 | |  |
| Family history of HCM (%) | 1 (25.0) | 0 (0.0) | 1 (4.8) | 0.35 | 0.171 | 0.699 | |  |
| Family history of SCD (%) | 0 (0.0) | 0 (0.0) | 0 (0.0) | NA | NA | NA | |  |
| Family history of stroke (%) | 0 (0.0) | 0 (0.0) | 3 (14.3) | NA | 0.42 | 0.484 | |  |
| Smoke (%) | 0 (0.0) | 1 (33.3) | 7 (33.3) | 0.212 | 0.174 | 1 | |  |
| Alcohol intake (%) | 0 (0.0) | 0 (0.0) | 3 (14.3) | NA | 0.42 | 0.484 | |  |
| NYHA Ⅲ/Ⅳ (%) | 0 (0.0) | 2 (100.0) | 6 (60.0) | 0.083 | 0.251 | 0.273 | |  |
| Episode of syncope (%) | 0 (0.0) | 0 (0.0) | 1 (4.8) | NA | 0.656 | 0.699 | |  |
| QTc (ms) | 478.33 ± 22.59 | 484.00 ± 85.07 | 453.33 ± 28.04 | 0.917 | 0.156 | 0.194 | |  |
| QTc >450 ms (%) | 3 (100.0) | 2 (66.7) | 11 (52.4) | 0.273 | 0.118 | 0.642 | |  |
| Atrial fibrillation (%) | 1 (25.0) | 0 (0.0) | 6 (28.6) | 0.35 | 0.884 | 0.285 | |  |
| Non-sustained ventricular tachycardia (%) | 1 (25.0) | 1 (33.3) | 1 (4.8) | 0.809 | 0.171 | 0.094 | |  |
| Atrio-ventricular block (%) | 0 (0.0) | 1 (33.3) | 0 (0.0) | 0.212 | NA | 0.007 | |  |
| Any arrhythmia (%) | 1 (25.0) | 2 (66.7) | 8 (38.1) | 0.27 | 0.617 | 0.348 | |  |
| Nonfatal stroke (%) | 1 (25.0) | 1 (33.3) | 4 (19.0) | 0.809 | 0.785 | 0.569 | |  |
| CAD (%) | 0 (0.0) | 1 (33.3) | 5 (23.8) | 0.212 | 0.275 | 0.722 | |  |
| Diabetes mellitus (%) | 0 (0.0) | 1 (33.3) | 2 (9.5) | 0.212 | 0.52 | 0.243 | |  |
| Max wall thickness (mm) | 23.50 ± 7.55 | 16.67 ± 2.08 | 18.95 ± 4.62 | 0.196 | 0.116 | 0.414 | |  |
| Max wall thickness ≥30 mm (%) | 1 (25.0) | 0 (0.0) | 0 (0.0) | 0.35 | 0.019 | NA | |  |
| IVS (mm) | 23.50 ± 7.55 | 16.67 ± 2.08 | 17.71 ± 5.18 | 0.196 | 0.068 | 0.736 | |  |
| LVPW (mm) | 12.50 ± 7.14 | 12.33 ± 3.21 | 12.00 ± 2.43 | 0.972 | 0.792 | 0.832 | |  |
| Apex (mm) | 15.25 ± 6.18 | 12.33 ± 4.04 | 11.19 ± 3.84 | 0.513 | 0.091 | 0.636 | |  |
| LAD (mm) | 38.00 ± 5.20 | 40.00 ± 2.65 | 39.52 ± 6.12 | 0.584 | 0.687 | 0.897 | |  |
| LVEDD (mm) | 45.67 ± 5.13 | 43.67 ± 4.62 | 46.67 ± 5.81 | 0.642 | 0.781 | 0.404 | |  |
| LVEF (%) | 65.00 ± 6.24 | 60.00 ± 2.65 | 61.00 ± 10.16 | 0.271 | 0.518 | 0.869 | |  |
| LVOTG ≥30 mm Hg (%) | 0 (0.0) | 1 (33.3) | 7 (33.3) | 0.212 | 0.174 | 1 | |  |
| E/A | 0.74 ± 0.09 | 1.21 ± 1.21 | 16.16 ± 41.15 | 0.544 | 0.531 | 0.543 | |  |
| E/E' | 15.72 ± 4.35 | 23.72 ± 7.33 | 16.77 ± 7.14 | 0.179 | 0.811 | 0.139 | |  |
| Values are N (%) or mean ± SD.  Abbreviations: *ALPK3tv*, HCM cases carrying *ALPK3* truncating variants; *ALPK3dmv*, HCM cases carrying *ALPK3* deleterious missense variants; *ALPK3nmv*, HCM cases carrying *ALPK3* neutral missense variants; SCD, sudden cardiac death; NYHA, New York Heart Association; CAD, coronary artery disease; IVS, interventricular septum; LVPW, left ventricular posterior wall; LAD, left atrial diameter; LVEDD, left ventricular end-diastolic dimension; LVEF, left ventricular ejection fraction; LVOTG, left ventricular outflow tract gradient. | | | | | | |  |  |
|  |  |  |  |  |  |  |  |  |
|  |  |  |  |  |  |  |  |  |
|  |  |  |  |  |  |  |  |  |
|  |  |  |  |  |  |  |  |  |
|  |  |  |  |  |  |  |  |  |

| **Supplementary Table 7. Genotype-inferred *ALPK3* expression-to-LV trait associations.** | | | |
| --- | --- | --- | --- |
| Trait | zscore | effect_size | pvalue |
| HCM | -4.483639718 | -1.244688811 | 7.34E-06 |
| LVEDV | 3.724770562 | 0.189374429 | 0.000195493 |
| LVEDVi | 3.14134535 | 0.197691527 | 0.001681736 |
| LVESV | 4.821837633 | 0.259965642 | 1.42E-06 |
| LVESVi | 4.397650001 | 0.279595293 | 1.09E-05 |
| LVEF | -4.364896104 | -0.271905534 | 1.27E-05 |
| SV | 1.79501072 | 0.0977913 | 0.072651992 |
| SVi | 1.197977474 | 0.076745238 | 0.230925787 |
| LVM | -2.587174816 | -2.711277893 | 0.009676648 |
| meanLVwallthickness | -5.193084662 | -0.229843578 | 2.07E-07 |
| LV_concentricity | -5.158663919 | -0.034030257 | 2.49E-07 |
| LV_radial_strain | -2.459635759 | -1.722583615 | 0.013907809 |
| LV_longitudinal_strain | -0.054367932 | -0.014869302 | 0.956642028 |
| LV_circumferential_strain | 3.967452188 | 1.102227873 | 7.26E-05 |
| Abbreviations: LV, left ventricle/ventricular; LVEDV, LV end-diastolic volume; LVEDVi, body-surface-area (BSA) indexed LVEDV; LVESV, LV end-systolic volume; LVESVi, BSA indexed LVESV; LVEF, LV ejection fraction; SV, stroke volume; SVi, BSA indexed SV; LVM, LV mass; meanLVwallthickness, mean LV wall thickness. | | | |
|  |  |  |  |
|  |  |  |  |
|  |  |  |  |
|  |  |  |  |
